# Claudin-5 relieves cognitive decline in Alzheimer’s disease mice through suppression of inhibitory GABAergic neurotransmission

**DOI:** 10.18632/aging.204029

**Published:** 2022-04-26

**Authors:** Ning Zhu, Meidan Wei, Linguang Yuan, Xiaodan He, Chunli Chen, Aimin Ji, Guozeng Zhang

**Affiliations:** 1 General Practice Center, The Seventh Affiliated Hospital, Southern Medical University, Foshan 528244, China; 2 Department of Pharmacy, The Third Affiliated Hospital of Southern Medical University, Guangzhou 510630, China; 3 College of Basic Medicine, Changsha Medical University, Changsha 410219, China; 4 Department of Pharmacy, The Seventh Affiliated Hospital, Southern Medical University, Foshan 528244, China; 5 Institute of Nursing and Health, School of Nursing and Health, Henan University, Kaifeng 475004, China

**Keywords:** Alzheimer’s disease, tight junction, claudin-5, learning and memory, GABAergic neurotransmission

## Abstract

Alzheimer’s disease (AD) is characterized by progressive cognitive decline, which is considered as the most common form of dementia in the elderly. Recently, it is suggested that impaired cerebrovascular function may precede the onset of AD. Claudin-5, which is the most enriched tight junction protein, has been reported to prevent the passage of damaging material at the blood-brain barrier. However, whether claudin-5 impacts AD has no direct evidence. We found a decrease level of claudin-5 in the hippocampus of AD and elder mice. And intravenous injection of claudin-5 improved learning and memory ability in these mice, while knockout of the protein led to impaired learning and memory and long-term potentiation in adult control mice. Furthermore, the effects of claudin-5 are mediated by suppressing inhibitory GABAergic neurotransmission. Our results suggest benefit effects of claudin-5 on learning and memory, which may provide a new treatment strategy for AD.

## INTRODUCTION

As a prevailing neurodegenerative disorder, AD is the main reason of dementia in the older people. By 2050, AD is estimated to affect about 14 million people in the United States of America [1, 2]. The main symptom of AD is deficits in cognition and behavior, which is relevant to atrophy and loss of neurons in the hippocampal region [3]. Except these, the main neuropathological features of AD also include synaptic loss, neuronal degeneration, accumulation of gliosis and amyloid beta (Aβ) [4, 5]. However, some investigators also observed the Aβ deposition in the cerebrovasculature [6–8]. Furthermore, in several types of vascular pathologies such as infarcts, the AD patients also found microbleeds, and white matter changes [9, 10]. These results make more and more interest in the role of cerebrovascular in AD pathogenesis.

Many investigations supported the role of neurovascular dysfunction in the onset and development of AD. Recently, brain imaging results indicated that cerebrovascular dysfunction may precede onset of neurodegenerative changes and cognitive decline both in AD patients and AD animal models [11–14]. Aβ deposition can lead to cytotoxic and pro-inflammatory occurrence in the vasculature of the AD brain, which contribute to the greater permeability of blood-brain barrier (BBB) [15–17]. Furthermore, cerebral amyloid angiopathy is reported to cause the disruption of the BBB [17]. And tau may also accelerate BBB deterioration [18–21]. Significantly, the BBB integrity was preserved when suppressed the tau expression [21]. So Aβ and tau may thus accelerate the BBB integrity loss, which probably also cause vascular dysfunction and AD [22].

As the major proteins of the BBB, claudins are critical for maintaining integrity of brain blood vessels [23, 24]. In humans, the superfamily of claudin includes more than 18 homologous proteins. Although claudin-1, claudin-3 and claudin-12 are expressed in endothelial cells of BBB as well, claudin-5 is the dominant claudin in these cells [25–27], which has been considered as the important factor in the endothelial permeability of the BBB [28, 29]. Reduced ability of the BBB to act against small molecules (<800 Da) was observed in claudin-5 deficient mice [29]. When exposed to high glucose, BBB permeability increased and claudin-5 expression levels decreased in brain microvascular endothelial cells [30]. These results provide a possibility that claudin-5 may mediated the BBB permeability that involved in AD process.

Therefore, in this study we want to know if claudin-5 affects the learning and memory ability in AD and elder mice, aiming to find a new method for treatment with AD.

## RESULTS

### Effects of intravenous injection of claudin-5 on learning and memory in AD mice

**Figure 1:**
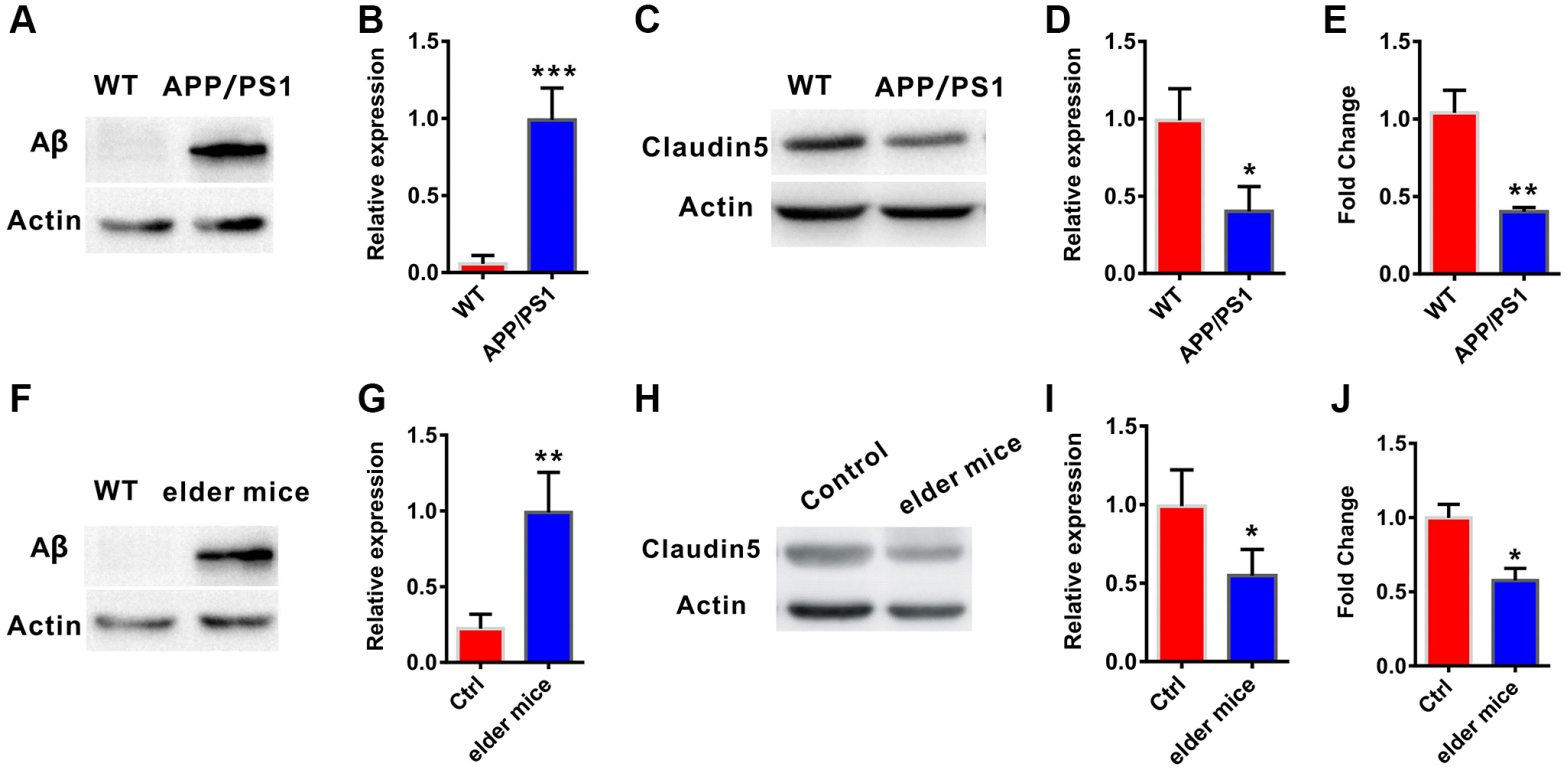
**Decreased claudin-5 level in AD and elder mice.** (**A**, **B**) The APP/PS1 mice exhibited increased Aβ protein level in hippocampal region (*n* = 4 per group; two-tailed Student’s *t*-test). (**C**, **D**) Decreased claudin-5 protein level in hippocampus of the APP/PS1 mice (*n* = 4 per group; two-tailed Student’s *t*-test). (**E**) Decreased claudin-5 mRNA level in hippocampus of APP/PS1 mice (*n* = 6 per group; two-tailed Student’s *t*-test). (**F**, **G**) The elder mice exhibited increased Aβ protein level in hippocampal region (*n* = 4 per group; two-tailed Student’s *t*-test, *P* = 0.001). (**H**, **I**) Decreased claudin-5 protein level in hippocampus of elder mice (*n* = 4 per group; two-tailed Student’s *t*-test). (**J**) Decreased claudin-5 mRNA level in hippocampus of elder mice (*n* = 6 per group; two-tailed Student’s *t*-test). Data show mean ± s.e.m. ^*^P < 0.05, ^**^P < 0.01, ^***^P < 0.001.

**Figure 2:**
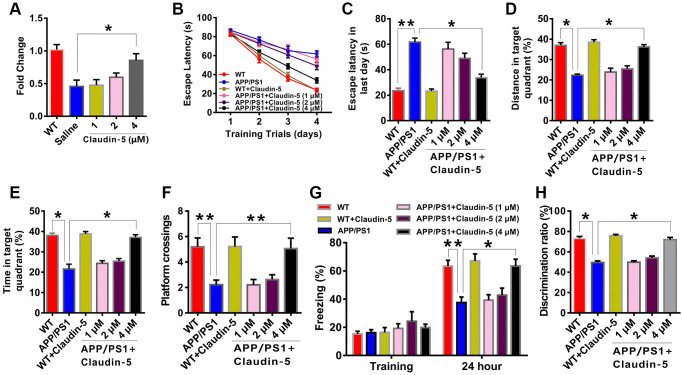
**Effects of intravenous injection of claudin-5 on learning and memory in APP/PS1 mice.** (**A**) Intravenous injection of claudin-5 restored the decreased claudin-5 mRNA level in hippocampus of APP/PS1 mice, which exhibited concentration-dependent (*n* = 6 per group; one-way ANOVA). (**B**–**F**) The ability to search the platform (**B** [4 consecutive days] and **C** [the last day]), swim distance in the target quadrant (**D**), spend time in the target quadrant (**E**), and cross the target quadrant number of times (**F**) in MWM between different group mice (*n* = 9–10 per group; repeated measures two-way ANOVA in **B**; one-way ANOVA in **C**–**F**). (**G**) The freezing time in contextual fear conditioning test between different group mice (*n* = 9–10 per group; one-way ANOVA). (**H**) The discrimination rate in NOR test between different group mice (*n* = 9–10 per group; one-way ANOVA). Data show mean ± s.e.m. ^*^P < 0.05, ^**^P < 0.01, ^***^P < 0.001.

To explore whether claudin-5 level was changed in AD, we firstly employed the APP/PS1 (a very commonly used AD model mice) mice aged at 10 months. Results from western blotting verified that, the amyloid-β (Aβ) protein level was increase in the hippocampus of APP/PS1 mice, compared with their control littermates (t = 0.863; *P* < 0.0001; Figure 1A, 1B). We then examined claudin-5 level from hippocampal tissues of these mice and found a decrease of claudin-5 both in protein (t = 1.263; *P* = 0.021; Figure 1C, 1D) and mRNA level (t = 1.013; *P* = 0.003; Figure 1E). To investigate if supplementation of claudin-5 had effects on cognitive ability of APP/PS1 mice, we intravenously injected different concentration of claudin-5 (1, 2, 4 μM) [31, 32] into the APP/PS1 mice. We found that intravenous injection of claudin-5 at 4 μM but not 1 or 2 μM restored decreased claudin-5 mRNA level (F_4,25_ = 35.845; *P* = 0.041; Figure 2A) without affecting other claudins (Supplementary Figure 1A) in APP/PS1 mice of the hippocampal region. We then employed the following hippocampus-dependent behaviors: MWM test [33], contextual fear conditioning test [34] and NOR test [35] in turn.

We firstly employed the MWM task to examine if intravenous injection of claudin-5 affected the hippocampus-involved learning and memory in APP/PS1 mice. After being trained for four consecutive sessions, control mice reached the hidden platform faster during the training. However, APP/PS1 mice exhibited impaired learning abilities (F_5,212_ = 66.342; *P* = 0.044; Figure 2B). We then explored the role of claudin-5 treatment on cognition and found that, the claudin-5-treated APP/PS1 mice concentration at 4 μM but not 1 or 2 μM found the hidden platform faster than claudin-5-treated WT mice (F_5,53_ = 34.274; *P* = 0.032; Figure 2C). In the probe trial, APP/PS1 mice reached the hidden platform with less time (F_5,53_ = 31.876; *P* = 0.033; Figure 2D) and distance (F_5,53_ = 32.856; *P* = 0.036; Figure 2E) in the target quadrant and less number of platform crossings, compared with WT mice (F_5,53_ = 31.945; *P* = 0.038; Figure 2F), and intravenous injection of 4 μM but not 1 or 2 μM claudin-5 can rescue these memory deficits in APP/PS1 mice (Figure 2B–2F). The average swimming speed (F_5,53_ = 18.745; *P* = 0.735; Supplementary Figure 2A) and locomotor activity (F_5,53_ = 17.465; *P* = 0.825; Supplementary Figure 2B) had no difference among these groups.

To further investigate if claudin-5 had a benefit effect, we performed the contextual fear conditioning test. Compared with WT mice, APP/PS1 mice exhibited less freezing time twenty-four hours after training. And intravenous injection of 4 μM but not 1 or 2 μM claudin-5 can relieve the behavior deficit (F_5,53_ = 43.835; *P* = 0.045; Figure 2G). In the NOR test, the claudin-5-treated group concentration at 4 μM but not 1 or 2 μM had a more preference to novel object than familiar one; however, APP/PS1 mice exhibited no preference (F_5,53_ =32.345; *P* = 0.036; Figure 2H). These results proved the fact that claudin-5 ameliorate the cognitive impairment in AD mice.

### Effects of intravenous injection of claudin-5 on cognition in elder mice

**Figure 3:**
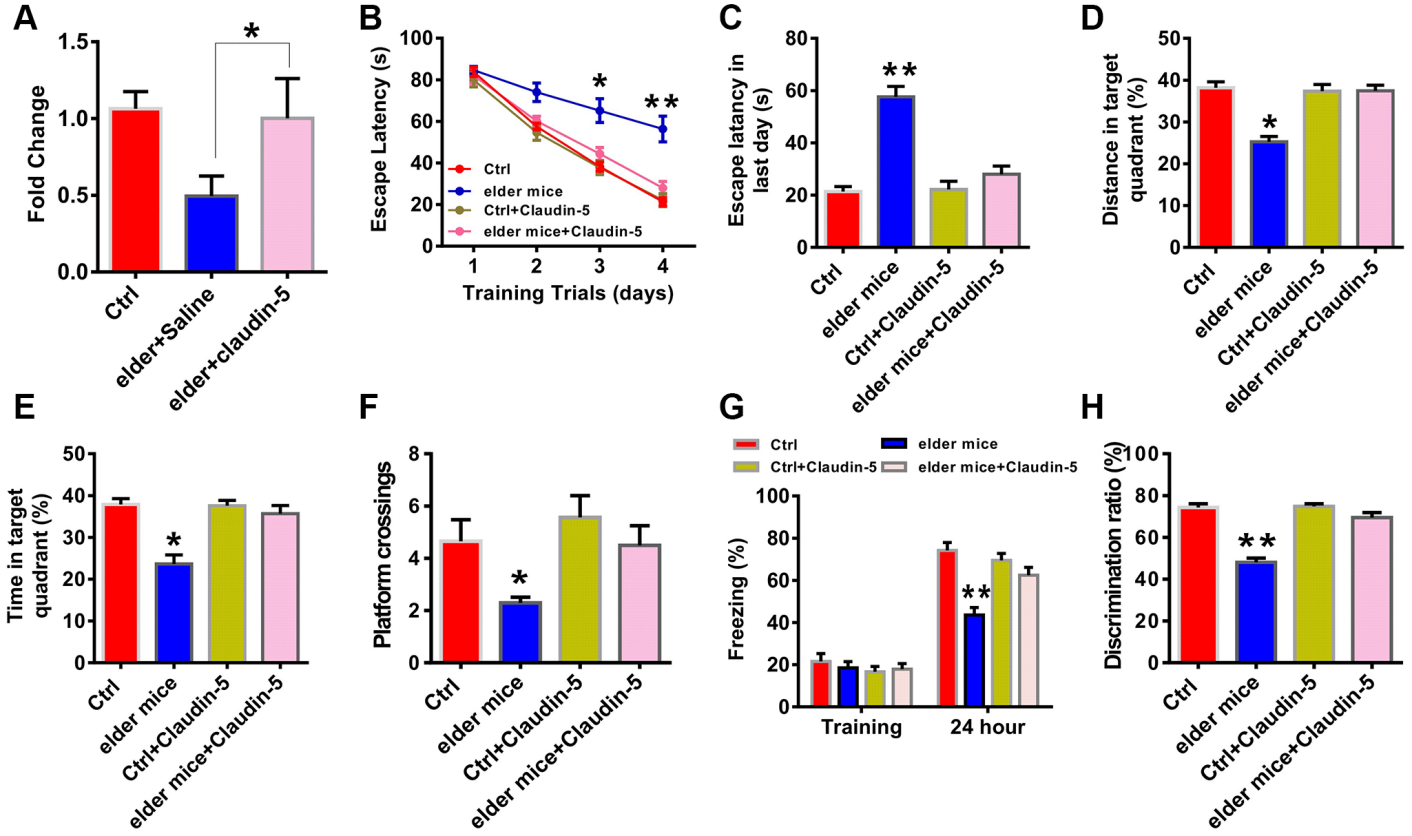
**Effects of intravenous injection of claudin-5 on learning and memory in elder mice.** (**A**) Intravenous injection of claudin-5 (4 μM) restored the decreased claudin-5 mRNA level in hippocampus of elder mice (*n* = 6 per group; one-way ANOVA). (**B**–**F**) The ability to search the platform (**B** [4 consecutive days] and **C** [the last day]), swim distance in the target quadrant (**D**), spend time in the target quadrant (**E**), and cross the target quadrant number of times (**F**) in MWM between different group mice (*n* = 8–10 per group; Repeated measures two-way ANOVA in **B**; one-way ANOVA in **C**–**F**). (**G**) The freezing time in contextual fear conditioning test between different group mice (*n* = 8–10 per group; one-way ANOVA). (**H**) The discrimination rate in NOR test between different group mice (*n* = 8–10 per group; one-way ANOVA). Data show mean ± s.e.m. ^*^P < 0.05, ^**^P < 0.01, ^***^P < 0.001.

To further explore whether claudin-5 had benefit effects, we employed the male C57BL/6J mice at 20 month years old, a developmental stage equivalent to the 65–75 years of human age [36] that accompanied by an increased Aβ protein level (t = 1.123; *P* = 0.001; Figure 1F, 1G). We first examined claudin-5 level and found that, both protein (t = 1.863; *P* = 0.036; Figure 1H, 1I) and mRNA (t = 1.564; *P* = 0.039; Figure 1J) level of claudin-5 was decreased in hippocampal region of elder mice, compared with adult mice (control, 2 months years old). We then investigated if supplementation of claudin-5 had effects on learning and memory in these mice. Intravenous injection of claudin-5 (4 μM) (F_2,15_ = 27.845; *P* = 0.021; Figure 3A) restored decreased mRNA level of claudin-5, without affecting other claudins (Supplementary Figure 1B). In animal behavior tests, we found that, while elder mice exhibited learning and memory impairment, intravenous injection of claudin-5 can ameliorate this impairment in elder mice (Figure 3B–3H). No differences were found in swimming speed or locomotor activity between elder and control mice (Supplementary Figure 2C, 2D). These results support a positive role of claudin-5 on cognition in elder mice.

### Effects of knockout of claudin-5 on cognition and LTP in adult mice

**Figure 4:**
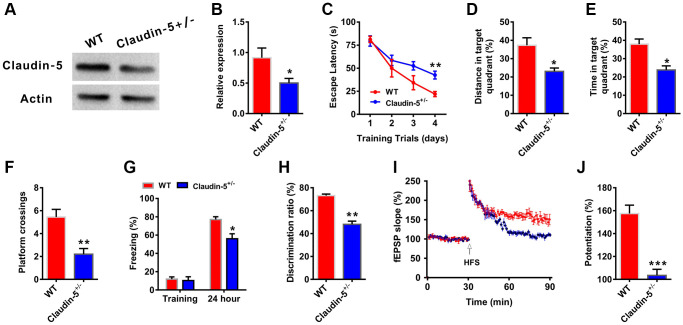
**Effects of knockout of claudin-5 on learning and memory and hippocampal LTP.** (**A**, **B**) The claudin-5^+/−^ mice exhibited decreased claudin-5 protein level in hippocampal region (*n* = 4 per group; two-tailed Student’s *t*-test). (**C**–**F**) The ability to search the platform (**C**), swim distance in the target quadrant (**D**), spend time in the target quadrant (**E**), and cross the target quadrant number of times (**F**) in MWM between WT and claudin-5^+/−^ mice (*n* = 9–10 per group; Repeated measures two-way ANOVA in **C**; two-tailed Student’s *t*-test in **D**–**F**). (**G**) The freezing time in contextual fear conditioning test between WT and claudin-5^+/−^ mice (*n* = 9–10 per group; two-tailed Student’s *t*-test). (**H**) The discrimination rate in NOR test between WT and claudin-5^+/−^ mice (*n* = 9–10 per group; two-tailed Student’s *t*-test). (**I**, **J**) Hippocampal CA1 LTP induction between WT and claudin-5^+/−^ mice (*n* = 6 per group; two-tailed Student’s *t*-test). Data show mean ± s.e.m. ^*^P < 0.05, ^**^P < 0.01, ^***^P < 0.001.

To further investigate whether there had relationship between claudin-5 and cognition, we employed the claudin-5 knockout mice. Because claudin-5^−/−^ mice died within ten hours after birth [29], we used claudin-5 heterozygote mice (claudin-5^+/−^, 8 weeks years old). Claudin-5 protein was significantly down-regulated in the hippocampus of claudin-5^+/−^ mouse, compared with their WT mouse (t = 0.723; *P* = 0.021; Figure 4A, 4B). And knockout of claudin-5 had no effect on the claudins expression profile (Supplementary Figure 1C). Then we performed the abovementioned behavioral tests and found that knocking out claudin-5 impaired learning and memory behaviors (Figure 4C–4H). While knockout of claudin-5 did not affect locomotor activity or swimming speed (Supplementary Figure 2E, 2F).

Long-term potentiation (LTP) in hippocampus is considered as the cellular mechanism of learning and memory [37]. We then recorded LTP to explore the cellular mechanism of claudin-5-induced learning and memory changes from WT mice and claudin-5^+/−^ mice. We found that, one train of high frequency stimulation (HFS)-induced LTP showed a significant difference between two groups (Figure 4I). And the slope of fEPSPs was 155.25 ± 1.75 and 102.68 ± 2.47 in WT group mice and claudin-5^+/−^ group mice, respectively (Figure 4J), which meant thatclaudin-5^+/−^ group animals exhibited impaired LTP compared with WT group mice.

### Effects of claudin-5 on glutamatergic neurotransmission

**Figure 5:**
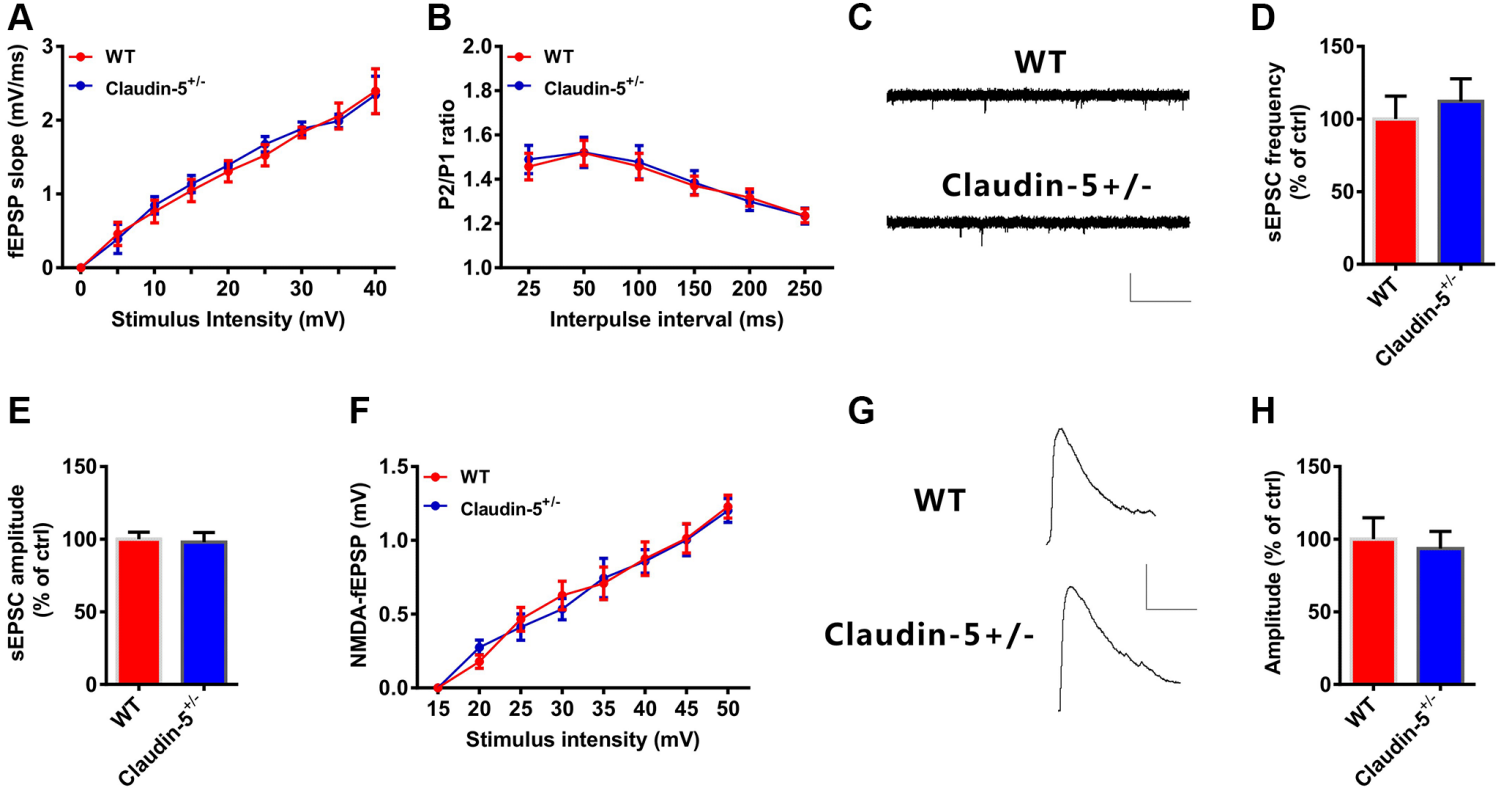
**Effects of knockout of claudin-5 on glutamatergic transmission in hippocampal slices.** (**A**) I-O curves in claudin-5^+/−^ mice and their control littermates (*n* = 6 per group; repeated measures two-way ANOVA). (**B**) PPF in claudin-5^+/−^ mice and their control littermates (*n* = 6 per group; repeated measures two-way ANOVA). (**C**–**E**) sEPSCs recording in claudin-5^+/−^ mice and their control littermates. Average sEPSC frequency (Hz) in (**D**) and amplitude in (**E**) were obtained (*n* = 9 per group; two-tailed Student’s *t*-test). Scale bars: 20 pA, 2 s. (**F**) NMDAR fEPSPs slopes in claudin-5^+/−^ mice and their control littermates (*n* = 6 slices/group; repeated measures two-way ANOVA). The fEPSPs were recorded in the presence of 20 μM CNQX and 0 nM Mg^2+^. (**G**, **H**) NMDA currents recording in claudin-5^+/−^ mice and their control littermates (*n* = 9 per group; two-tailed Student’s *t*-test). Scale bars: 50 pA, 100 ms. Data show mean ± s.e.m.

LTP induction is predominantly modulated by glutamatergic synaptic transmission [38]. However, we did not observe detectable changes in basal synaptic transmission in the Input-Output curves (Figure 5A), and presynaptic release in PPF (Figure 5B) after claudin-5 knocking out. Moreover, knockout of the claudin-5 did not affect either the amplitude or the frequency of the spontaneous excitatory postsynaptic currents (sEPSCs, Figure 5C–5E). These results indicate that claudin-5 have no effect on alpha-amino-3-hydroxy-5-methyl-4-isoxazolepropionic acid (AMPA) receptor (AMPAR) -mediated synaptic transmissions. To explore whether claudin-5 mediates N-methyl-D-aspartate (NMDA) receptor (NMDAR)-mediated synaptic transmissions, we recorded fEPSPs using 6-cyano-7-nitroquinoxaline-2,3-dione (CNQX, 20 μM) to block AMPAR and Mg^2+^-free buffer to relieve the NMDAR block. However, knockout of the claudin-5 did not affect the slopes of NMDAR-mediated fEPSPs as the Input-Output curves completely overlapped between WT and claudin-5^+/−^ mice (Figure 5F). We then recorded NMDAR-mediated EPSCs in hippocampal pyramidal neurons and did not observe change in NMDAR-EPSCs after claudin-5 knockout (Figure 5G, 5H). Above all, these results indicate that claudin-5 does not affect glutamatergic transmission in hippocampal brain region.

### Claudin-5 mediated synaptic plasticity by promoting GABA-mediated synaptic responses

**Figure 6:**
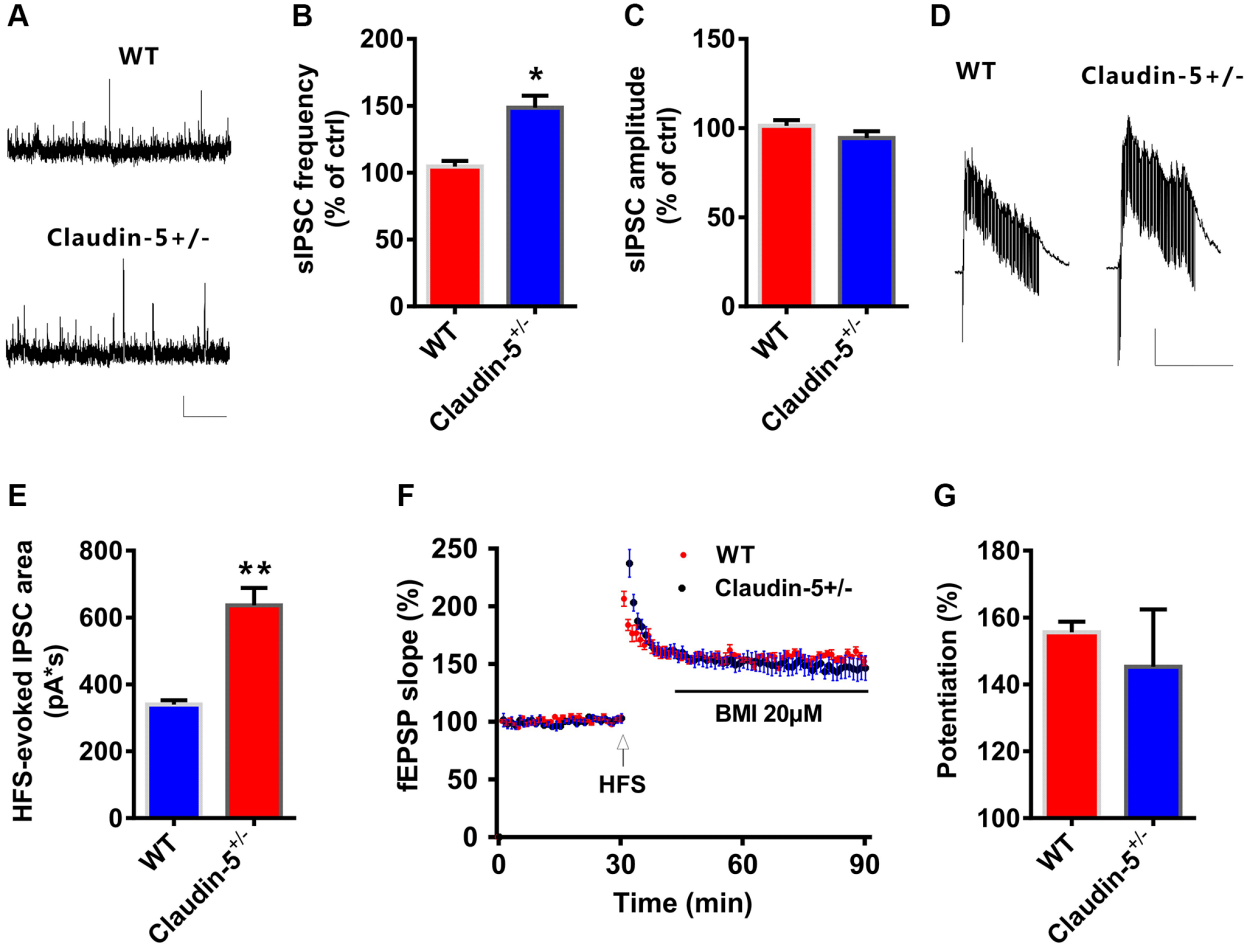
**Claudin-5 modulated GABAergic transmission to mediate synaptic plasticity.** (**A**–**C**) Effects of claudin-5 knockout on sIPSCs frequency and amplitude (*n* = 9 per group; two-tailed Student’s *t*-test). Scale bars: 20 pA, 2 s. (**D**, **E**) HFS-evoked IPSCs during HFS in hippocampal CA1 neurons in claudin-5^+/−^ mice and their control littermates (*n* = 9 cells from 4 mice; two-tailed Student’s *t*-test). Scale bars: 200 pA, 1 s. (**F**, **G**) The GABA_A_ receptor antagonist BMI blocked impaired effects of claudin-5 knockout on LTP (*n* = 6; two-tailed Student’s *t*-test). Data show mean ± s.e.m. ^*^P < 0.05, ^**^P < 0.01, ^***^P < 0.001.

The aforementioned results showed that claudin-5 had no effect on glutamatergic transmission. To further investigate if claudin-5 affected the GABAergic transmission, we recorded the spontaneous inhibitory postsynaptic currents (sIPSCs). We found that, knockout of claudin-5 led to increase the frequency of sIPSCs with no effect on the amplitude (Figure 6A–6C). Moreover, knockout of claudin-5 also increased the HFS-evoked feedforward IPSCs (Figure 6D, 6E), which suggested that impaired LTP in claudin-5 knockout mice probably via its enhancing GABAergic transmission. Furthermore, *ex vivo* results indicated that the GABAa receptor selectively antagonist bicuculline (BMI) can prevent the LTP impairment in claudin-5^+/−^ mice (Figure 6F, 6G), which supported an involvement of GABAergic mechanism that mediated impaired LTP in claudin-5^+/−^ mice.

## DISCUSSION

The main findings in our study were as follows. First, decreased claudin-5 protein and mRNA level was found in AD and elder mice. Second, intravenous injection of claudin-5 can improve cognitive ability in AD and elder mice. Third, knockout of claudin-5 disturbed learning and memory behaviors and LTP induction. And lastly, GABAergic neurotransmission was involved in claudin-5 mediated LTP. Altogether, our results suggested benefit effects of claudin-5 on cognitive ability for AD and provided new treatment strategy for it.

AD is the main cause of dementia which was typically presented with a progressive loss of cognitive function and memory [39–41]. AD is an incurable and chronic neurodegenerative disease which is estimated to impact 5.4 million people in the United States of America [42]. Cerebrovascular and neuronal dysfunctions are symptoms of AD which lead to a progressive loss of cognitive functions [43]. More recently, studies from epidemiological, clinical imaging and pharmacotherapy have suggested that changes in vascular have a critical role in early AD pathogenesis [11–14]. Hypoperfusion of cerebral and impaired clearance of Aβ across the BBB may contribute to the onset and development of AD. Cerebral blood flow decrease negatively influences the proteins synthesis that was required for learning and memory, which may lead to AD eventually. Impaired Aβ clearance from the brain may also cause its accumulation, which is associated with cognitive decline and is also one of the features of AD.

At the BBB claudin-5 is the dominant claudin which is expressed in wide types of organs, such as the brain, lung, breast, kidney, liver, gastrointestinal tract, reproductive organs, pancreas and skin [44]. Claudin-5 loss alone is sufficient to lead to functional BBB changes [45]. Expression of claudin-5 can be modulated by kinds of upstream signaling pathways, such as cyclic adenosine mono-phosphate (cAMP) and protein kinase A (PKA) [46], which are important to LTP and learning and memory. However, as we all know, there is no evidence to show whether claudin-5 mediate learning and memory in AD. In our study, we detected a decrease mRNA and protein level of claudin-5 in hippocampus of APP/PS1 and elder mice. And intravenous injection of claudin-5 can reverse the impaired learning and memory behaviors in these mice. These results, to our knowledge, firstly provide a direct evidence of the positive function of claudin-5 on learning and memory of AD mice. As mentioned above, claudin-5 is express predominantly on brain endothelial cells but also in other cells of neurovascular units [25–27]. Recently microglial cells were indicated as potential source of claudin-5 for BBB reparation [47]. So, it is very important to identify the source of claudin-5 in regulating learning and memory ability as well LTP in AD or elder mice, which can better understand the relationship between claudin-5 and learning and memory. We will explore these issues in our future studies. It is also worth noting that, decreased claudin-5 expression in AD mice may affect the functional status of BBB. So if we want to explore whether claudin-5 affects learning and memory through mediating the functional status of BBB, we should determine the BBB integrity and degree of BBB damage in all experimental groups in our future study.

LTP induction is modulated mainly by glutamatergic synaptic transmission [38]. However, in our study, claudin-5 knockout had no effect on I-O curves, PPF or sEPSCs. However, knockout of claudin-5 increased the sIPSCs frequency and the HFS-evoked IPSC amplitude. At the same time, blocking the GABA_A_ receptors using BMI prevented the LTP impairment in claudin-5 knockout mice. So, we suppose that claudin-5-enhanced GABAergic neurotransmission may decrease postsynaptic depolarization [48], which thereby leading to impaired LTP induction.

In conclusion, our study indicated the role of the claudin-5 in the ability of cognition in AD and elder mice, which was mediated by GABAergic transmission. So our results may provide new insight for treatments with learning and memory disability in AD.

## MATERIALS AND METHODS

### Animals

All experimental animals were housed in groups of 3–5 per cage with free access to food and water on a 12:12-h light/dark cycle (lights on at 8:00 am and off at 8:00 pm) in a room temperature at 21–25°C. All mouse protocols were approved by the Institutional Animal Care and Use Committee at Southern Medical University [49]. Claudin-5^+/−^ mice were generated by the Nanjing Biomedical Research Institute of Nanjing University (Nanjing, China).

### Open field test

As reported in previous study [50], mice were firstly placed in the center of a chamber (40 × 40 × 30 cm), then we monitored the movement for each mice during five min with a help of an overhead camera. Images of the path travelled during 5 min were automatically obtained, and distance travelled was calculated with a VersaMax animal behavioral monitoring system (Omnitech Electronics, Nova Scotia, Canada).

### Morris water maze (MWM) test

As reported in previous study [51], Training trials were conducted during four consecutive days after group housing. A 10 cm diameter platform was placed below the water (1 cm). We started the quadrant randomized, and each mice performed under the same order. The time that the mice reach the hidden platform and the swimming speed were recorded. Animals can stay on the platform for 30 s once they reached the platform. Or we will guide it to the platform if they did not able to find the platform within 120 s. On day 5 we removed the platform and monitored the total distance and time spent in the target quadrant for 60 s, as well as the numbers of platform crossings.

### Contextual fear conditioning test

As reported in previous study [50], after habituated to the room firstly, each mouse subjected a 3 min exploration period. During training, each mouse exposed to four trials (consisting of a 30 s, 80 dB tone-conditioned stimulus followed by a 1 s 0.75 mA footshock, 80 s interstimulus interval) in the conditioning chamber A. The mice were kept in the chamber A for 1 min after the last footshock and then were returned to their home cages. Twenty-four hours later, to test contextual fear conditioning, the mice were placed into the chamber A for 3 min. The percentage of freezing time was measured using automated motion detection software (FreezeFrame and FreezeView).

### Novel object recognition (NOR) test

As reported in previous study [51], on first day each mouse was placed in the empty arena for 5 min to habituate it. On second day (the familiarization session), we placed two identical objects 5cm away from the walls. And mice were able to freely explore each object until total exploration time reached to 20 s or stop the experiment when a 10-min-period is finish. On third day, we place two identical objects (one is familiar objects and the other is novel object) in the arena. We will stop the experiment when total exploration time reached to 20 s or when a 10-min-period is finish. The discrimination ratio equals to time spent exploring novel object divided by total exploring time.

### Electrophysiological recordings

All experimental protocols were performed in accordance with previous studies [50]. Mice were anaesthetized with ethyl ether and their brains were rapidly removed and chilled in ice-cold oxygenated modified artificial cerebrospinal fluid (ACSF) containing (in mM) 220 sucrose, 2.5 KCl, 2.5 MgSO_4_, 1.3 CaCl_2_, 26 NaHCO_3_, 1 NaH_2_PO_4_, and 10 mM glucose. Coronal hippocampal slices (300 μm) were prepared using a vibratome (VT-1200S, Leica, Germany), and the slices were incubated in a holding chamber that contained oxygenated ACSF containing (in mM) 120 NaCl, 1.2 NaH_2_PO_4_, 2.5 KCl, 2.0 CaCl_2_, 26 NaHCO_3_, 2.0 MgSO_4_, and 10 glucose at 34°C for 30 min and then maintained at room temperature for at least 1 hour before recording. LTP was induced by one train HFS at 100-Hz. The PPF was measured using pairs of stimuli with inter-pulse intervals between 20–200 ms.

Whole-cell patch clamp recordings were performed by using an upright microscope (Nikon, ECLIPSE FN1), which was equipped with a 40× water-immersion lens and infrared-sensitive camera (DAGE-MTI, IR-1000E). The Pipettes fabricated from filamented borosilicate glass capillary tubes (inner diameter, 0.84 μm) by using a horizontal puller (Sutter Instruments, P-97). For sEPSC recording, neurons were held at a potential of -70 mV. Glass pipettes resistance was typically 3–7 MΩ filled with internal solution containing (in mM) 130 K-gluconate, 10 HEPES buffer, 20 KCl, 0.3 Na-GTP, 4 Mg-ATP, 0.2 EGTA and 10 disodium phosphocreatine (pH 7.2 with KOH, 280−300 mOsm), For sIPSC recording, the holding potentials were −70 mV filled with internal solution containing (in mM) 35 K-gluconate, 2 EGTA, 100 KCl, 5 NaCl, 10 HEPES, 0.1 NaGTP, 5 QX-314, 2 MgATP (pH 7.3, 280−300 mOsm) in the presence of 1 mM kynurenic acid (KA). NMDAR current was recorded in the presence of 20 μM CNQX to block AMPAR. For HFS-evoked IPSC recording, neurons were held at a potential of 0 mV filled with internal solution containing (in mM): 115 cesium methanesulphonate, 10 HEPES, 20 CsCl, 10 sodium phosphocreatine, 2.5 MgCl_2_, 5 QX-314, 0.4 Na_3_GTP, 0.6 EGTA and 4 Na_2_-ATP (pH 7.3, 285 mOsm).

### Western blotting

Western blotting was performed in accordance with previous studies [52]. Brain tissue homogenates isolated from the whole hippocampus were prepared, and the protein concentration was determined with a BCA Protein Assay Kit (Thermo, #23227). The samples were then run on 12% SDS-PAGE gels and transferred to PVDF membranes (Millipore, MA, USA), which were then blocked with 5% nonfat milk at room temperature for 1 h and then incubated overnight at 4°C with a primary antibody [anti-claudin-5 (1:1000, ab172968, Abcam, Cambridge, MA, USA) and anti-Aβ (1:1000, ab62658, Abcam, Cambridge, MA, USA)] at 4°C. The membranes were washed three times in TBST and incubated with HRP-conjugated secondary antibody [monoclonal mouse anti-β-actin (1:1000; Bostor, China) at room temperature for 1 hour. For semiquantitative analysis, the protein bands were detected with an imaging system (Universal Hood II, Bio-Rad, Segrate, Italy) and quantitatively analyzed using Image Lab software. The optical density (OD) of each band normalized to the OD of the internal control.

### Real-time quantitative PCR (qRT-PCR)

Using the Universal qRT-PCR master mix, the qRT-PCR was performed for the indicated genes (Takara) using a Stratagene Mx3000P thermal cycler. The primers used for qRT-PCR were as follows:

**Table:** 

**Gene**	**Sense**	**Antisense**	**Size (bp)**
Claudin-1	GCAGAAGATGAGGATGGCTGT	CCTTGGTGTTGGGTAAGAGGT	253
Claudin-2	GCCATGATGGTGACATCC AGT	TCAGGCACCAGTGGTGAGTAG	218
Claudin-3	GGACTTCTACAACCCCGTGGT	AGACGTAGTCCTTGCGGTCGT	230
Claudin-4	CAAGGCCAAGACCATGATCGT	GCGGAGTAAGGCTTGTCTGTG	246
Claudin-5	TTTCTTCTATGCGCAGTTGG	GCAGTTTGGTGCCTACTTCA	247
Claudin-6	GGTGCTCACCTCTGGGATTGT	GCAGGGGCAGATGTTGAGTAG	267
Claudin-7	CTCGAGCCCTAATGGTGGTCT	CCCAGGACAGGAACAGGAGAG	326
Claudin-8	CCGTGATGTCCTTCTTGGCTTTC	CTCTGATGATGGCATTGGCAACC	176
Claudin-9	GGTACACTGGGCACCTGTGAT	GCTTCGACCGGCTTAGAACTG	312
Claudin-10	CTGTGGAAGGCGTGCGTTA	CAAAGAAGCCCAGGCTGACA	132
Claudin-11	CTGATGATTGCTGCCTCGGT	ACCAATCCAGCCTGCATACAG	243
Claudin-12	AGTCACTGCTCCCGTCATACC	TTCTGAATCTGGCCCAAGTCT	250

Gene expression was normalized to the expression of GAPDH as a housekeeping gene and calculated using the ΔΔCT method.

### Statistical analysis

All results are presented as the mean ± standard error (SEM). Using GraphPad Prism version 8.2 (GraphPad Software, Inc., USA) to perform the statistical analyses using ANOVA and Student’s *t* test unless otherwise specified. Statistical significance was defined as ^*^p < 0.05, ^**^p < 0.01, and ^***^p < 0.001.

## Supplementary Materials

Supplementary Figures
