# Supplementary material for: Claudin-5 relieves cognitive decline in Alzheimer’s disease mice through suppression of inhibitory GABAergic neurotransmission
Source: Aging (Albany NY). 2022 Apr 26;14(8):3554–68. doi: 10.18632/aging.204029 (PMC9085235; doi:10.18632/aging.204029)
Supplement: Supplementary Figures [file aging-14-204029-s001.pdf]

SUPPLEMENTARY FIGURES

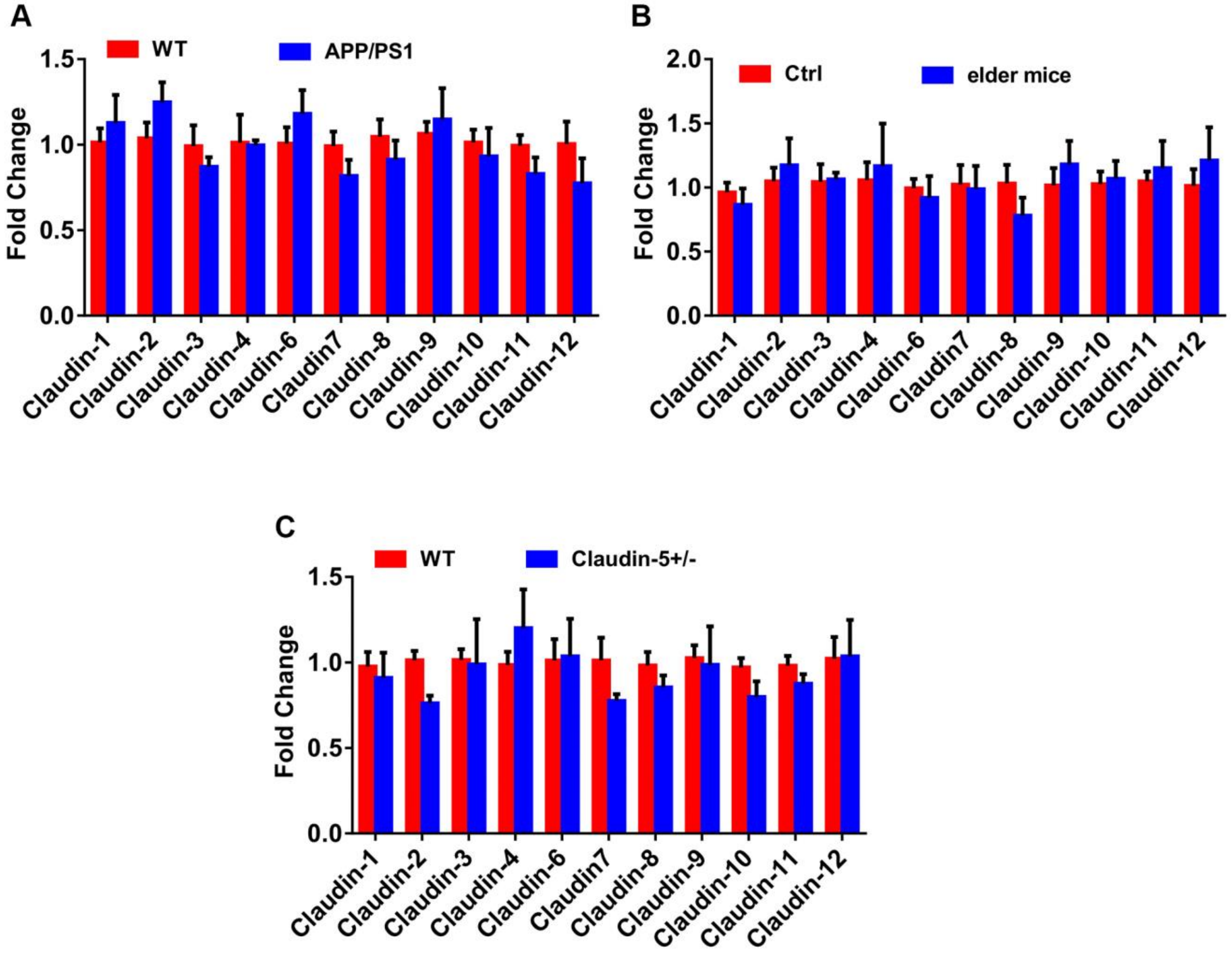

Supplementary Figure 1. The claudins expression profile in the hippocampus of mice after different treatment. (**A**) Claudins mRNA level in the hippocampus of APP/PS1 mice and their control littermates (*n* = 6 per group; two-tailed Student's *t*-test). (**B**) Claudins mRNA level in the hippocampus of elder mice and adult control mice (*n* = 6 per group; two-tailed Student's *t*-test). (**C**) Claudins mRNA level in the hippocampus of claudin-$5^{+/-}$ mice and their control littermates (*n* = 6 per group; two-tailed Student's *t*-test). Data show mean ± s.e.m.

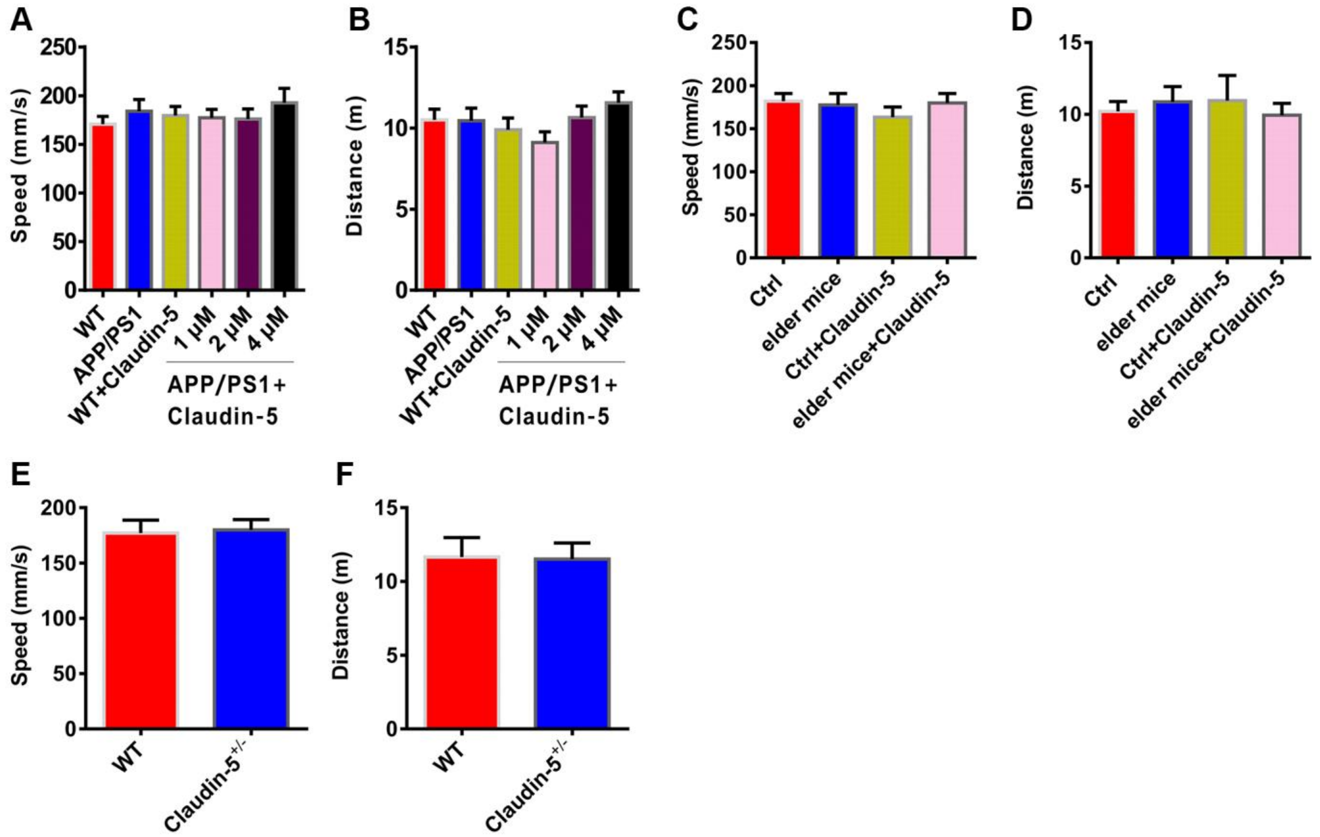

Supplementary Figure 2. The swimming speed and locomotor activity after different treatment. (**A**, **B**) Swimming speed in the MWM (**A**, *n* = 9–10 per group; one-way ANOVA) and locomotor activity in the open field test (**B**, *n* = 9–10 per group; one-way ANOVA) for APP/PS1 mice with or without intravenous injection of claudin-5. (**C**, **D**) Swimming speed in the MWM (**C**, *n* = 8–10 per group; one-way ANOVA) and locomotor activity in the open field test (**D**, *n* = 8–10 per group; one-way ANOVA) for elder mice with or without intravenous injection of claudin-5. (**E**, **F**) Swimming speed in the MWM (**E**, *n* = 9–10 per group; two-tailed Student's *t*-test) and locomotor activity in the open field test (**F**, *n* = 9–10 per group; two-tailed Student's *t*-test) for WT and claudin-$5^{+/-}$ mice. Data show mean ± s.e.m.
